# Supplementary figures and images for: Genetic structure and knockdown resistance (kdr) mutations in Aedes albopictus (Skuse) (Diptera: Culicidae): Implications for dengue fever transmission in southeastern China
Source: PLoS One. 2025 Mar 26;20(3):e0320200. doi: 10.1371/journal.pone.0320200 (PMC11940661; doi:10.1371/journal.pone.0320200)

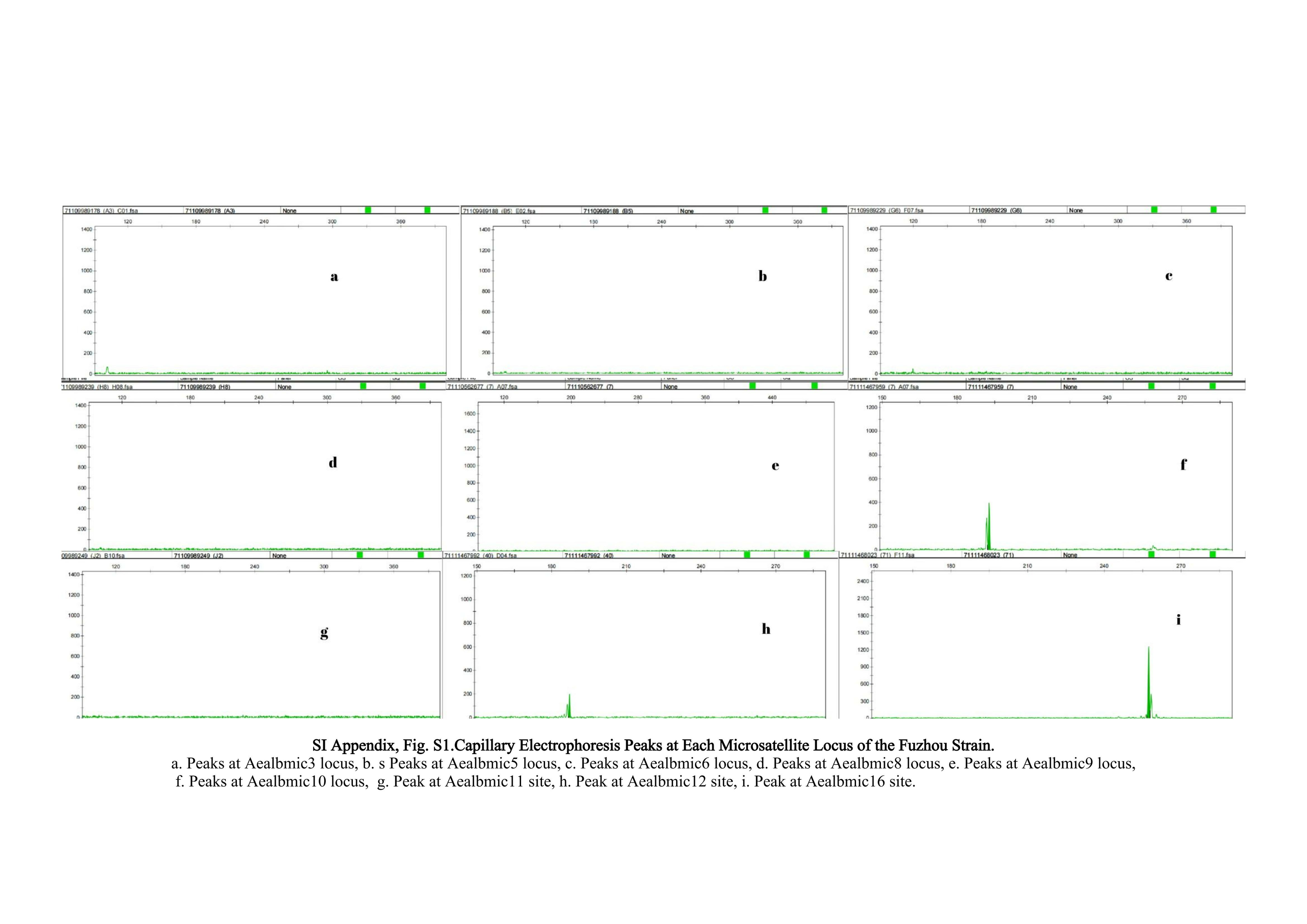

Supplement: S1 Fig — (TIF) [file pone.0320200.s001.tif]

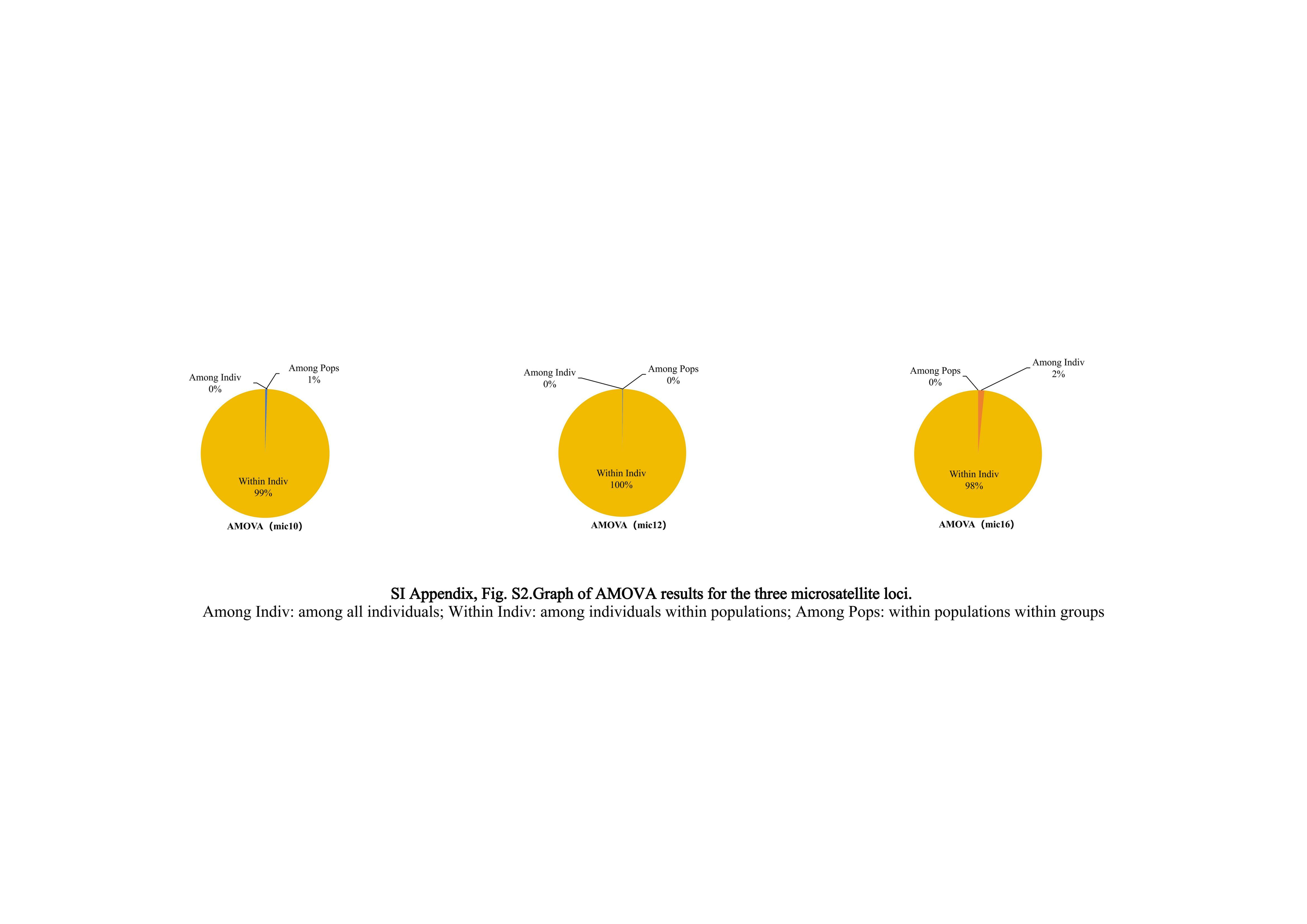

Supplement: S2 Fig — (TIF) [file pone.0320200.s002.tif]
