# Supplementary material for: Genetic structure and knockdown resistance (kdr) mutations in Aedes albopictus (Skuse) (Diptera: Culicidae): Implications for dengue fever transmission in southeastern China
Source: PLoS One. 2025 Mar 26;20(3):e0320200. doi: 10.1371/journal.pone.0320200 (PMC11940661; doi:10.1371/journal.pone.0320200)
Supplement: S1 Table — (DOCX) [file pone.0320200.s003.docx]

**Table S1.** Primer sequences for PCR amplification of microsatellite loci (upstream primer with M13 sequence tag (5'− CCCTCATAGTTAGCGTAACG− 3') (Wei 2021)

| Primer | Forward primer | Reverse primer |
| --- | --- | --- |
| SSRmic3 | ACCATACAGCCTGGAGTTCG | GGGGTTGTGTGAATTGTCGT |
| SSRmic5 | AACCCATCGAACACAGAAGG | GTACGGTTGACTCGCTGTGA |
| SSRmic6 | GATGGTCCGTATTTGGGTTG | ATCTTCACTCATCCGCCATC |
| SSRmic8 | TTGTTGTTCGGTTGTTGTTTG | CGGGTTCCAACTATGTACGA |
| SSRmic9 | GCGATGACAGTGGAACAAGA | GCTTGGCAGGGAACAAATTA |
| SSRmic10 | ATCGCCTTCACTCTTCTTCG | CCAATCCTGAGCCGTACATT |
| SSRmic11 | CTCTGCGTTCCGGTTCTATC | AGGCAACCTCTCGAATGAAA |
| SSRmic12 | AGAGCCCTCGAAAAGAGAGC | AGCACTCATTCTTGGCTTGG |
| SSRmic16 | GGAATGGTTCCCTGGCTAAT | CCAACTCCGAAGAAGCCATA |
