# Supplementary material for: Genetic structure and knockdown resistance (kdr) mutations in Aedes albopictus (Skuse) (Diptera: Culicidae): Implications for dengue fever transmission in southeastern China
Source: PLoS One. 2025 Mar 26;20(3):e0320200. doi: 10.1371/journal.pone.0320200 (PMC11940661; doi:10.1371/journal.pone.0320200)
Supplement: Attachment — Submitted filename: Review PONE.pdf [file pone.0320200.s005.pdf]

Genetic Structure and Knockdown Resistance (*kdr*) Mutations in *Aedes albopictus* (Skuse) (Diptera: Culicidae): Implications for Dengue Fever Transmission in Southeastern China

This study looks for the interrelation of genetic structure and resistance status in Aedes albopictus with dengue cases. The study has substantial data to be published. However, the authors are required to explain more on the results part and also deepen their discussion to support the results. Here is my specific comments:

1. Italic- *kdr*
2. Require high resolution for all figures.
3. Introduction: No coherent and smooth transition between paragraphs. For example, paragraph 2 discusses the genetic population, and suddenly, paragraph 3 discusses resistance.
4. The introduction is too shallow. Requires more pieces of literature.
5. Line 58: Male or female mosquitoes? What stage is it?
6. Line 61: What do you mean by fed turtle?
7. Line 63: “The F1-F2 L3-L4”- Rephrase to make it clear.
8. Line 64: FS strain. What is FS strain? Is it a susceptible strain? How long has it been cultured?
9. Line 73: No Table 1 on the sequence and primer was provided.
10. Line 88-89: State the 5-7 gradient concentration that you have used.
11. Line 93: “treated group should be corrected”- are you doing the correction on your treated group? What is the mortality of your control set? Determine why you need to make the Abbot correction.
12. Line 100-101: There were 8 localities mentioned. And why only 5 localities were reported in this section? Give an explanation why only 5 were chosen.
13. Line 102-103: add the primers sequence in the manuscript.
14. Line 103: Give the name brand for the Master mix.
15. Line 137-139: “The NA of per population ranged from 2 to 6, the NE varied from 1.52 to 5.00, and the expected heterozygosity (He) spanned from 0.34 to 0.80(Table 2)”. Explain more about these figures/values.
16. Line 147-152: Your results are just reporting the range of values. Please give an explanation/meaning of the results.
17. 157: The study has LC50 values, but no explanation of this part was included in the results. Please include it.
18. For the RR value, which strain becomes the reference strain? Is it a susceptible strain?
19. Lack of discussion. Expand your discussion to discuss why the genetic divergence showed high differentiation. How did the gene flow happen, and why is it not substantial?
20. Expand the deltamethrin resistance discussion. What is the reason for the resistance? Which domain of resistance? Any supporting study that is related to your finding? Did any other study in China find the resistances in points I1532 and F1534?
21. Lack of discussion on genetic indicators and dengue incidence- expand it.
22. Line 244- “similar capacities”. Please explain further what you mean by this sentence.

23. Line 251: ‘Appropriate dosage of insecticides’. What do you mean by this? The insecticide will be applied according to the recommended dose by the manufacturer in the field.

Thanks
