# Supplementary material for: Genetic structure and knockdown resistance (kdr) mutations in Aedes albopictus (Skuse) (Diptera: Culicidae): Implications for dengue fever transmission in southeastern China
Source: PLoS One. 2025 Mar 26;20(3):e0320200. doi: 10.1371/journal.pone.0320200 (PMC11940661; doi:10.1371/journal.pone.0320200)
Supplement: Attachment — Submitted filename: Response to Reviewers.docx [file pone.0320200.s007.docx]

**Title of the Manuscript:** **Genetic Structure and Knockdown Resistance (*kdr*) Mutations in *Aedes albopictus* (Skuse) (Diptera: Culicidae): Implications for Dengue Fever Transmission in Southeastern China**

**Manuscript Number:** PONE-D-24-53355

Dear Editor-in-Chief Cosme and Reviewers,

We are deeply appreciative of the opportunity to revise our manuscript entitled “**Genetic Structure and Knockdown Resistance (*kdr*) Mutations in *Aedes albopictus* (Skuse) (Diptera: Culicidae): Implications for Dengue Fever Transmission in Southeastern China**”. We extend our sincere thanks to the reviewers for their time and the meticulous attention they have dedicated to evaluating our work. Their constructive feedback has been invaluable in enhancing the clarity and scholarly depth of our manuscript. In response, we have meticulously revised various sections, including:

- The introduction to the experimental methods, ensuring a clear background context.
- The description of the experimental methods, with corrections for accuracy.
- The presentation of experimental results, with improvements for better clarity and understanding.
- The description of the outcomes, now more detailed and comprehensive.

We have addressed each point raised by the reviewers and provided a detailed, point-by-point response below. Additionally, we have incorporated the necessary revisions into the manuscript, with changes highlighted for easy reference.

**Reviewer #1:**

1. This is a very interesting paper. I think it is worth publishing, but it needs major revisions and clarifications. Lines 17: Abbreviations that first appear should have full names.

*Reply:* *Thank you very much for your positive feedback and for recognizing the paper as interesting and worthy of publication. We are pleased to hear that you find value in our work and are eager to address the points you've raised to ensure the paper meets the high standards necessary for publication.*

*In response to your comment regarding the abbreviation on Line 17, we have made the necessary revision. The abbreviation "VGSC" now appears with its full form "voltage-gated sodium channel " upon first mention, providing clarity for readers who may not be familiar with the term. This change has been implemented to enhance the accessibility and understanding of our research.*

2. Lines 33, 40… References should be cited in accordance with journal requirements (and the reference section at the end of the article)

*Reply:* *Thank you for pointing out the mistake, we have carefully reviewed and updated the references to ensure they are formatted correctly and consistently throughout the manuscript.*

3. Lines 59-60: Level 1 provinces? Are the words used accurately and are the grammar correct? The entire article needs to be checked for sentence grammar.

*Reply:* *Thank you for your insightful comments and suggestions. We appreciate your attention to detail and are committed to ensuring that our manuscript is of the highest quality. In response to your specific comment about the term "Level 1 provinces" and the need to check the entire article for sentence grammar:*

*1、We have replaced "Level 1 provinces" with "first-level province"(Line 61-62)" to more accurately reflect the administrative hierarchy in China. This term is more commonly used and understood in the context of Chinese administrative divisions.*

*2、We have conducted a thorough review of the entire article to ensure that all sentences are grammatically correct and clear. We have used both manual proofreading and grammar-checking tools to identify and correct any potential issues.*

4. Lines 60-66: No details about sampling were provided...How many spatial points (households?) per location? How can you be sure you sampled representative subset of a population at a given location? (e.g sampling members of few families)?

*Reply:* *Thank you for your valuable comments and suggestions. We appreciate your attention to the details of our sampling methodology and are committed to providing a clear and comprehensive explanation.*

*Here are the specific details:*

*Sampling Locations and Spatial Points:*

*We conducted our sampling across multiple locations, with each location being a distinct geographical area. The number of spatial points (households) sampled per location varied based on the population density and the expected variability in the mosquito population. On average, we sampled 30 adult Aedes albopictus mosquitoes per location, divided into 3 groups of 10 mosquitoes each. Each group was sampled from different districts within the city to ensure a representative subset of the population. This approach was chosen to account for potential spatial heterogeneity within each location.*

*Ensuring Representativeness:*

*To ensure that our sample was representative of the broader population at each location, we employed a stratified sampling design. We first identified homogeneous subgroups (strata) within each location based on environmental and demographic factors known to influence mosquito populations. A simple random sample was then drawn from each subgroup to ensure that all segments of the population were adequately represented in our study. This method helps to reduce variance in the estimates and ensures that our findings are more generalizable to the entire population.*

*Avoiding Bias:*

*We were mindful of the potential for bias in our sampling and took steps to minimize it. For instance, we avoided convenience sampling methods that might have led to over-representation of households near main roads or other easily accessible areas. Instead, we used a random route method, where enumerators followed a predetermined path to select households, ensuring that all areas within a location had an equal chance of being included in the sample. This approach helps to avoid frame coverage errors and ensures that our sample is not biased towards households with specific characteristics.*

*This approach allowed us to account for potential non-responses and ensure that we achieved a representative sample size.*

*We have included these details in the revised manuscript (Line 61-Line 65)to provide a more comprehensive understanding of our sampling methodology. We believe that these revisions will enhance the clarity and robustness of our research.*

5. L215-218: "signifying... suggesting... indicated...” What are these conclusions based on? Judgment criteria? Is it supported by the literature?

*Reply:* *Thank you for your insightful comments and suggestions. We appreciate your attention to the details of our conclusions and are committed to providing a clear and comprehensive explanation.*

*In response to your comment regarding the conclusions in lines 215-218, we have revised the manuscript to include more information about the basis of these conclusions. Here are the specific details:*

*Basis of Conclusions: The conclusions in lines 215-218 are based on a combination of empirical data and statistical analysis. We have conducted a thorough analysis of the genetic data collected from the Aedes albopictus populations, focusing on the presence and frequency of kdr mutations (F1534S and F1534L) and their correlation with genetic diversity indices. We used a combination of molecular techniques, including PCR and sequencing, to identify the presence of these mutations in the sampled populations. The data were then analyzed using statistical methods to determine the significance of the observed correlations.*

*Judgment Criteria: The judgment criteria for these conclusions were based on established statistical thresholds and biological relevance. Specifically, we used a p-value threshold of 0.05 to determine statistical significance in our analyses. This threshold is commonly used in biological research to ensure that the observed effects are not due to random chance. Additionally, we considered the biological significance of the observed genetic variations and their potential impact on the mosquito's resistance to insecticides. This was assessed by comparing the mutation frequencies across different populations and evaluating their potential to affect the mosquito's fitness and survival in the presence of insecticides.*

*we have supplemented detailed analysis and explanations in the previous results(Line 168-179) in the revised manuscript to provide a more comprehensive understanding of the basis of our conclusions. We believe that these revisions will enhance the clarity and robustness of our research.*

6. L226-227: Your results can only indicate that there is no correlation between kdr mutations and the genetic indicators displayed by the three loci you selected, but it cannot mean that the mutation of kdr does not affect the genetic structure. The conclusion should not be amplified.

*Reply:* *Thank you for your insightful comments and suggestions. We appreciate your attention to the details of our conclusions and are committed to providing a clear and comprehensive explanation. We have already revised the expression in this part, and we hope to conduct in-depth research in the future to determine whether there will be an impact on the genetic structure. The specific revisions are as follows(Line 263-266):*

*There was no significant difference in the correlation between the mutation rates of I1532 and F1534 and the genetic indicators, indicating that the kdr mutations have not yet had an impact on the genetic level of the population at the mic10, mic12, and mic16 loci. Further in-depth research is needed to determine whether these mutations have an impact on the genetic structure of the population.*

*We have included these details in the revised manuscript to provide a more comprehensive understanding of the basis of our conclusions. We believe that these revisions will enhance the clarity and robustness of our research*

7. L243-244: “The study confirmed that…” The entire study does not have data and result on the transmission ability of the virus, so this conclusion cannot be drawn. The conclusion of the article has the problem of excessive amplification. The author is requested to review and revise it, and the conclusion should be inferred based on your own research results.

*Reply: Thank you for your insightful comments and suggestions. We appreciate your attention to the details of our conclusions and are committed to providing a clear and comprehensive explanation, we have revised the manuscript to avoid any over-amplification of the results and provided a more detailed explanation of this conclusion(Line 293-296).*

**Reviewer #2:**
1. Add to the discussion, add some recent valuable references.

*Reply:* *Thank you for your suggestions. We have added some references from the past five years to the discussion section.* *The specific literature is as follows.*

*Li Y, Zhou G, Zhong D, Wang X, Hemming-Schroeder E, David RE, et al. Widespread multiple insecticide resistance in the major dengue vector Aedes albopictus in Hainan Province, China. Pest management science. 2021;77(4):1945-53. Epub 2020/12/11. doi: 10.1002/ps.6222.*

*Zheng X, Zheng Z, Wu S, Wei Y, Luo L, Zhong D, et al. Spatial heterogeneity of knockdown resistance mutations in the dengue vector Aedes albopictus in Guangzhou, China. Parasites & vectors. 2022;15(1):156. Epub 2022/05/04. doi: 10.1186/s13071-022-05241-7.*

*Rui-ling Z, Guang-qin Y, Xiao-qian P, De-zhen M, Ai-hua Z, Zhong Z. Genetic diversities of different geographical populations of Aedes albopictus based on mitochondrial gene COI. Chinese Journal of Zoonoses. 2017;33(04):316-20.*

2.Improve picture quality.

*Reply: Thank you for your comment regarding the picture quality in our manuscript. We understand the importance of high-quality images for the clarity and impact of our research. We have utilized the Adobe Illustrator to upscale and enhance the quality of our images. We have re-uploaded the enhanced images to our manuscript, and we believe that these improvements will significantly enhance the visual quality and impact of our research*

3.Improve table quality.

*Reply: Thank you for your comment regarding the table quality in our manuscript. We understand the importance of clear and well-presented data for the clarity and impact of our research.* *We have ensured that all tables present data in a clear and precise manner. Each table is organized with clear headers and appropriate column widths to facilitate easy reading and comparison.* *We have also used shading or font formatting to highlight important data points or patterns, enhancing readability.*

4.Strengthen the quality of foreword writing.

*Reply:* *Thank you for your valuable feedback regarding the quality of the foreword writing in our manuscript. We understand the importance of a well-crafted foreword in setting the stage for our research and engaging the reader.* *We have revised the introduction section, adding some references to make it more comprehensive. Moreover, we have paid attention to improving the coherence between paragraphs(Line 39-42).*

*The references we added are as follows:*

*Sabir MJ, Al-Saud NBS, Hassan SM. Dengue and human health: A global scenario of its occurrence, diagnosis and therapeutics. Saudi journal of biological sciences. 2021;28(9):5074-80. Epub 2021/09/02. doi: 10.1016/j.sjbs.2021.05.023.*

*Shan W, Yuan H, Chen H, Dong H, Zhou Q, Tao F, et al. Genetic structure of Aedes albopictus (Diptera: Culicidae) populations in China and relationship with the knockdown resistance mutations. Infectious diseases of poverty. 2023;12(1):46. Epub 2023/05/06. doi: 10.1186/s40249-023-01096-x.*

*Zhang Y, Zang C, Pan X, Gong M, Liu H. Population genetic diversity analysis of the dengue vector Aedes albopictus in China %J China Tropical Medicine. 2024;24(8):914-9.*

*Nakajima Y, Wepfer PH, Suzuki S, Zayasu Y, Shinzato C, Satoh N, et al. Microsatellite markers for multiple Pocillopora genetic lineages offer new insights about coral populations. Scientific reports. 2017;7(1):6729. Epub 2017/07/29. doi: 10.1038/s41598-017-06776-x.*

**Reviewer#3**

This study looks for the interrelation of genetic structure and resistance status in Aedes albopictus with dengue cases. The study has substantial data to be published. However, the authors are required to explain more on the results part and also deepen their discussion to support the results.

*Reply:* *Thank you for your positive feedback and constructive comments on our study. We are pleased that you find the data substantial and worthy of publication. We are committed to enhancing the clarity and depth of our results and discussion sections to better support our findings.*

1. Italic-kdr

*Reply:* *We have ensured that "kdr" is consistently italicized throughout the manuscript to adhere to the standard formatting for genetic terms.*

2. Require high resolution for all figures.

*Reply: Thank you for your comment regarding the picture quality in our manuscript. We understand the importance of high-quality images for the clarity and impact of our research. We have utilized the Adobe Illustrator to upscale and enhance the quality of our images. For Figure 1, it was created using ARCGIS. The map information is publicly available and there is no copyright protection issue. We have re-uploaded the enhanced images to our manuscript, and we believe that these improvements will significantly enhance the visual quality and impact of our research*

3. Introduction: No coherent and smooth transition between paragraphs. For example, paragraph 2 discusses the genetic population, and suddenly, paragraph 3 discusses resistance.

*Reply:* *Thank you for your valuable feedback on the introduction section of our manuscript. We appreciate your attention to the flow and coherence of our writing and are committed to making the necessary improvements to enhance the readability and logical progression of our introduction. We have added a connecting sentence between paragraphs 2 and 3 to ensure a smooth transition and to clarify the logical flow of our introduction(Line 39- 42). This sentence now explicitly links the discussion of the genetic population of Aedes albopictus to the subsequent discussion of resistance, highlighting the importance of understanding both aspects in the context of dengue fever control.*

*Moreover, we have revised the introduction section, adding some references to make it more comprehensive.*

*The references we added are as follows:*

*Sabir MJ, Al-Saud NBS, Hassan SM. Dengue and human health: A global scenario of its occurrence, diagnosis and therapeutics. Saudi journal of biological sciences. 2021;28(9):5074-80. Epub 2021/09/02. doi: 10.1016/j.sjbs.2021.05.023.*

*Shan W, Yuan H, Chen H, Dong H, Zhou Q, Tao F, et al. Genetic structure of Aedes albopictus (Diptera: Culicidae) populations in China and relationship with the knockdown resistance mutations. Infectious diseases of poverty. 2023;12(1):46. Epub 2023/05/06. doi: 10.1186/s40249-023-01096-x.*

*Zhang Y, Zang C, Pan X, Gong M, Liu H. Population genetic diversity analysis of the dengue vector Aedes albopictus in China %J China Tropical Medicine. 2024;24(8):914-9.*

*Nakajima Y, Wepfer PH, Suzuki S, Zayasu Y, Shinzato C, Satoh N, et al. Microsatellite markers for multiple Pocillopora genetic lineages offer new insights about coral populations. Scientific reports. 2017;7(1):6729. Epub 2017/07/29. doi: 10.1038/s41598-017-06776-x.*

4. The introduction is too shallow. Requires more pieces of literature.

*Reply:* *Thank you for your suggestion of enriching the introduction section. The citations we have added are as follows:*

*Sabir MJ, Al-Saud NBS, Hassan SM. Dengue and human health: A global scenario of its occurrence, diagnosis and therapeutics. Saudi journal of biological sciences. 2021;28(9):5074-80. Epub 2021/09/02. doi: 10.1016/j.sjbs.2021.05.023.*

*Shan W, Yuan H, Chen H, Dong H, Zhou Q, Tao F, et al. Genetic structure of Aedes albopictus (Diptera: Culicidae) populations in China and relationship with the knockdown resistance mutations. Infectious diseases of poverty. 2023;12(1):46. Epub 2023/05/06. doi: 10.1186/s40249-023-01096-x.*

*Zhang Y, Zang C, Pan X, Gong M, Liu H. Population genetic diversity analysis of the dengue vector Aedes albopictus in China %J China Tropical Medicine. 2024;24(8):914-9.*

*Nakajima Y, Wepfer PH, Suzuki S, Zayasu Y, Shinzato C, Satoh N, et al. Microsatellite markers for multiple Pocillopora genetic lineages offer new insights about coral populations. Scientific reports. 2017;7(1):6729. Epub 2017/07/29. doi: 10.1038/s41598-017-06776-x.*

5. Line 58: Male or female mosquitoes? What stage is it?

*Reply:* *Thank you for your careful reading. We have added more detailed explanations:* *Male and female adult mosquitoes were collected for genetic analysis to explore their association with dengue incidence and resistance mutations.(Line 66)*

6. Line 61: What do you mean by fed turtle?

*Reply: Thank you for pointing out the mistake. We have already corrected it:* *Larvae were fed turtle food to the adult stage in the laboratory.(Line 69)*

7. Line 63: “The F1-F2 L3-L4”- Rephrase to make it clear

*Reply: We have written out its full name to make the meaning more explicit.* *The specific revisions are as follows:* *The third and fourth larvae(L3-L4) stage of the first and second filial generation(F1-F2) were specifically used for pyrethroid susceptibility testing.(Line 69-71)*

8. Line 64: FS strain. What is FS strain? Is it a susceptible strain? How long has it been cultured?

*Reply: Thank you for your detailed and insightful comments on our manuscript. We appreciate your attention to the specifics of our study.* *The FS strain refers to a specific isolate of Aedes albopictus that has been used in our study. It is indeed a susceptible strain, which means it does not exhibit resistance to the insecticides tested in our experiments. This strain was chosen as a control to compare the resistance levels of other populations. It was generously donated by the research group led by Professor Chen Xiaoguang. The FS strain has been in culture for a significant period since 1980s, which allows us to maintain consistent genetic characteristics and ensure the reliability of our experimental results.*

9. Line 73: No Table 1 on the sequence and primer was provided.

*Reply: Thank you for your comment regarding the missing Table 1 on the sequence and primer used in our study. We apologize for this oversight and have now included the necessary information in the revised manuscript. We have already corrected it:* *The microsatellite sequences and their primers are detailed in the accompanying Table S1 (Line 79-80)*

10. Line 88-89: State the 5-7 gradient concentration that you have used.

*Reply: Thank you for your comment regarding the gradient concentration used in our study. We appreciate your attention to the details of our experimental design and are committed to providing a clear and comprehensive explanation.*

*In our study, we used a 5-7 gradient concentration to assess the impact of insecticides on Aedes albopictus populations before the formal experiment. These concentrations were chosen to cover a wide range of potential exposure levels, from low to high, to accurately assess the dose-response relationship. Then five gradient concentrations were used. For details, please refer to SI Appendix, Excel. S1 Bioassay Experiments.*

11. Line 93: “treated group should be corrected”- are you doing the correction on your treated group? What is the mortality of your control set? Determine why you need to make the Abbot correction.

*Reply:* *The supplementary instructions on data processing are as follows: If more than 10% of the control larvae pupate in the course of the experiment, the test should be discarded and repeated. If the control mortality is between 5% and 20%, the mortalities of treated groups should be corrected according to Abbott’s formula.(Line 95-98)*

12. Line 100-101: There were 8 localities mentioned. And why only 5 localities were reported in this section? Give an explanation why only 5 were chosen.

*Reply:* *Thank you for your comment regarding the selection of localities in our study. We appreciate your attention to the details of our experimental design and are committed to providing a clear and comprehensive explanation.*

*In our study, we initially identified 8 localities for sampling. However, we reported data from only 5 localities in the specific section you mentioned. The reason for this selection was that the data from these 5 localities were of high quality and completeness, allowing for robust statistical analysis. We ensured that the samples from these localities were sufficient in size and representative of the local populations*

*We have included these details in the revised manuscript to provide a clearer understanding of the rationale behind our locality selection. The samples from 8 sampling sites were used for the research on population genetics and resistance. Among them, "Mutation Analysis of Two Representative Alleles of the VGSC Gene" mainly focused on five populations in Fujian Province, namely the SM, FZ, PT, QZ, and ZZ strains.(Line109-110)*

13. Line 102-103: add the primers sequence in the manuscript.

*Reply:* *The genomic DNA was directly extracted from adult Aedes albopictus specimens and used as a template.* *The primers sequences were according to* (*Xu J, Bonizzoni M, Zhong D, Zhou G, Cai S, Li Y, et al. Multi-country Survey Revealed Prevalent and Novel F1534S Mutation in Voltage-Gated Sodium Channel (VGSC) Gene in Aedes albopictus. PLoS neglected tropical diseases. 2016;(1935-2735 (Electronic)). doi: 10.1371/journal.pntd.0004696.*

14. Line 103: Give the name brand for the Master mix.

*Reply: Thank you for your comment regarding the Master Mix used in our study. We appreciate your attention to the details of our experimental setup and are committed to providing a clear and comprehensive explanation. In our study, we used the TaKaRa Taq™ Version 2.0for our PCR reactions. We have included this information in the revised manuscript to provide a clearer understanding of the experimental materials used in our study(Line 114). We believe that these revisions will enhance the clarity and robustness of our research.*

15. Line 137-139: “The NA of per population ranged from 2 to 6, the NE varied from 1.52 to 5.00, and the expected heterozygosity (He) spanned from 0.34 to 0.80(Table 2)”. Explain more about these figures/values.

*Reply:* *Thank you for your comment regarding the figures and values mentioned in lines 137-139. We appreciate your attention to the details of our genetic analysis and are committed to providing a clear and comprehensive explanation. We have included these details in the revised manuscript to provide a clearer understanding of the genetic indices and their significance in our study (Line 151-158):*

*Among them, at the mic10 locus, the NA (6), NE (4.17) and I (1.61) of the Zhangzhou population (ZZ) are all higher than those in other regions; at the mic12 locus, the NA (6) and NE (5.00) of the Fuzhou population(FZ), Guangzhou population(GZ) and Zhejiang population(ZJ) are higher than those in other regions, and the I (1.70) of the Fuzhou population(FZ) and Zhejiang population(ZZ) is higher than that in other regions; at the mic16 locus, the NA (5), NE (3.13) and I (1.36) of the Guangzhou population(GZ) are all higher than those in other regions, and the diversity of the above-mentioned populations is relatively rich. The strain richness levels in the remaining regions are similar. The Ho of all populations sampled this time is greater than He, indicating a rich heterozygote.*

16. Line 147-152: Your results are just reporting the range of values. Please give an explanation/meaning of the results.

*Reply: Thank you for your comment regarding the explanation and meaning of the results reported in lines 147-152. We appreciate your attention to the details of our findings and are committed to providing a more comprehensive interpretation of our data. We have included these interpretations in the revised manuscript to provide a more comprehensive understanding of our findings(Line 168-179):*

*Considering that for all the populations mentioned above, the observed heterozygosity (Ho) is greater than the expected heterozygosity (He) at the three loci, it can be deduced that the mosquitoes from all sampled locations all result from out - breeding. This leads to a high degree of heterozygosity, which is consistent with the analysis results of the number of alleles (NA), effective number of alleles (NE), and Shannon's information index (I). Generally, an Fst value less than 0.05 is considered to indicate mild population differentiation. When the Fst value ranges from 0.05 to 0.15, it suggests moderate population differentiation. And if the Fst value is between 0.15 and 0.25, it implies high - level population differentiation. The genetic differentiation among populations was measured by Fst. The values ranged from 0.07 to 0.18, falling within the range of 0.05 - 0.25, indicating a moderate to high level of genetic differentiation among the four groups. The estimated Nm values fall between 1.13 and 3.25, suggesting that the populations can withstand the inter - population genetic differentiation resulting from genetic drift and that gene flow exists among them. (shown in Table 3).*

17. The study has LC50 values, but no explanation of this part was included in the results. Please include it.

*Reply: Thank you for your comment regarding the LC50 values in our study. We appreciate your attention to the details of our results and are committed to providing a clear and comprehensive explanation. We have included these interpretations in the revised manuscript:*

*LC50 represents the concentration of deltamethrin required to cause mortality in 50% of the larvae.(Line 191-192)*

*Therefore, it can be seen that the Ae. albopictus of the SM population was relatively sensitive to deltamethrin.(Line 195-196)*

18. For the RR value, which strain becomes the reference strain? Is it a susceptible strain?

*Reply:* *Thank you for your question regarding the reference strain used for calculating the Resistance Ratio (RR) values in our study. We appreciate your attention to this important detail. In our study, the reference strain used for calculating RR values is indeed a susceptible strain. Specifically, we used the FS strain as the reference strain.*

19. Lack of discussion. Expand your discussion to discuss why the genetic divergence showed high differentiation. How did the gene flow happen, and why is it not substantial?

*Reply: Thank you for your comment regarding the discussion on genetic divergence and gene flow in our study. We appreciate your suggestion to expand our discussion to provide a deeper understanding of why the genetic divergence showed high differentiation and why gene flow is not substantial.*

*We have expanded our discussion in the revised manuscript to include these points and provide a more comprehensive interpretation of our findings.(Line 252-257): The high genetic differentiation observed in our study is likely a result of a combination of geographic isolation, selective pressures, and genetic drift. It might also be caused by factors such as the bottleneck effect in the population's history. While gene flow can counteract genetic divergence, it was not substantial enough in our study to prevent high differentiation. Understanding these processes is crucial for designing effective vector control strategies, especially in regions where genetic divergence might influence the effectiveness of insecticides.*

*(Song G, Feng i, Jinna W, Yuyan W, Juan H, Zhenyu G. Genetic diversity of Aedes albopictus populations in the coastal areas of southern China, using the microsatellite markers. Chinese Journal of Epidemiology. 2019;40(8):992-6.)*

20. Expand the deltamethrin resistance discussion. What is the reason for the resistance? Which domain of resistance? Any supporting study that is related to your finding? Did any other study in China find the resistances in points I1532 and F1534?

*Reply: Thank you for your suggestion to expand the discussion on deltamethrin resistance in our study. We appreciate your interest in understanding the underlying mechanisms and supporting evidence for the observed resistance patterns. The resistance to deltamethrin in Aedes albopictus populations is multifaceted, involving both target-site mutations (kdr) and metabolic resistance mechanisms.*

*The F1534S and I1532T mutations in the VGSC gene are key contributors to resistance, with their distribution and impact varying by region. Understanding these mechanisms is crucial for developing effective vector control strategies, especially in regions where resistance is prevalent.*

*We have expanded our discussion in the revised manuscript to include these points and provide a more comprehensive interpretation of our findings (Line 269-275):* *Yijie Li et al. evaluated the multiple insecticide resistances (IR) in urban Ae.albopictus populations. In the Ae.albopictus populations in Guangzhou, they found that the F1534S and F1534L mutations were significantly associated with deltamethrin resistance. Besides, long - term insecticide selection and genetic isolation of Ae.albopictus populations seem to be the reasons for the continuous, patchy distribution of kdr mutant alleles. The small - scale spatial heterogeneity in the distribution and frequency of kdr mutations may have important implications for vector control operations and insecticide resistance management strategies.*

*(Li Y, Zhou G, Zhong D, Wang X, Hemming-Schroeder E, David RE, et al. Widespread multiple insecticide resistance in the major dengue vector Aedes albopictus in Hainan Province, China. Pest management science. 2021;77(4):1945-53. Epub 2020/12/11. doi: 10.1002/ps.6222. PubMed PMID: 33301644; PubMed Central PMCID: PMCPMC7986907.*

*Zheng X, Zheng Z, Wu S, Wei Y, Luo L, Zhong D, et al. Spatial heterogeneity of knockdown resistance mutations in the dengue vector Aedes albopictus in Guangzhou, China. Parasites & vectors. 2022;15(1):156. Epub 2022/05/04. doi: 10.1186/s13071-022-05241-7. PubMed PMID: 35505385; PubMed Central PMCID: PMCPMC9066732.)*

21. Lack of discussion on genetic indicators and dengue incidence- expand it.

*Reply: Thank you for your suggestion to expand the discussion on the relationship between genetic indicators and dengue incidence in our study. We appreciate your interest in understanding this complex interplay more deeply. We have expanded our discussion in the revised manuscript to include these points and provide a more comprehensive interpretation of our findings (Line 281-287):* *The Ae.albopictus populations in the southeastern coastal areas of China exhibit a moderate to high level of differentiation. Frequent gene flow is an important factor in preventing differentiation among populations. Meanwhile, gene flow also endows Ae.albopictus populations in different geographical regions with similar virus - carrying capabilities. Additionally, as areas with intensive human exchanges, the coastal regions face an increasing pressure from imported cases of mosquito - borne infectious diseases such as dengue fever. This insight underscores the complexity of dengue transmission, highlighting that it cannot be solely attributed to the genetic traits of Ae.albopictus.*

*(Rui-ling Z, Guang-qin Y, Xiao-qian P, De-zhen M, Ai-hua Z, Zhong Z. Genetic diversities of different geographical populations of Aedes albopictus based on mitochondrial gene COI. Chinese Journal of Zoonoses. 2017;33(04):316-20.)*

22. Line 244- “similar capacities”. Please explain further what you mean by this sentence.

*Reply: Thank you for your comment regarding the phrase "similar capacities" in line 244. We appreciate your attention to the clarity and precision of our language. To address your request for further explanation, we have revised the sentence to provide a clearer and more detailed description.*

*Revised Sentence:*

*We have replaced the phrase "similar capacities" with "similar virus-carrying capacities" to more accurately convey our intended meaning. The revised sentence now reads: "* *Our study confirmed that these populations exhibit similar virus-carrying capacities, indicating a potential for widespread transmission of dengue fever."(Line296-298)*

*Detailed Explanation:*

*The term "similar virus-carrying capacities" refers to the ability of Aedes albopictus populations to harbor and transmit the dengue virus. In our study,* *we observed that the genetic diversity and population structure of Aedes albopictus in the sampled locations suggest that these populations have comparable capabilities to carry and transmit the dengue virus. This observation is based on the genetic similarity and the absence of significant barriers to gene flow among these populations.*

23.Line 251: ‘Appropriate dosage of insecticide’. What do you mean by this? The insecticide will be applied according to the recommended dose by the manufacturer in the field.

*Reply:* *Thank you for your comment regarding the term "appropriate dosage of insecticide" in line 251. We appreciate your attention to this important aspect of our study. To address your query, we have expanded our explanation as follows:*

*The concept of "appropriate dosage of insecticide" is central to our study, emphasizing the need for tailored and strategic insecticide use to control Aedes albopictus populations effectively while minimizing resistance development. We have revised the manuscript to include this detailed explanation and to highlight the importance of this approach in managing insecticide resistance（Line309-311）.*

We trust that the modifications made to our manuscript now meet the standards of *PLOS ONE* and that it is deemed suitable for publication. We are grateful for the opportunity to contribute to the advancement of knowledge in our field and are enthusiastic about the potential impact of our findings.

Sincerely,

Li-hua Xie

lhxie@fjmu.edu.cn
